# Global burden of sepsis: a systematic review

**DOI:** 10.1186/cc14101

**Published:** 2015-03-16

**Authors:** C Fleischmann, A Scherag, NK Adhikari, CS Hartog, T Tsaganos, P Schlattmann, DC Angus, K Reinhart

**Affiliations:** 1 Jena University Hospital, Jena, Germany; 2 University of Toronto, Canada; 3 University of Athens, Greece; 4 University of Pittsburgh, PA, USA

## Introduction

Sepsis is a global healthcare challenge. However, comprehensive information on sepsis morbidity and mortality across the world is scarce. We aimed to estimate the global burden of sepsis and to identify knowledge gaps based on available evidence from observational epidemiological studies.

## Methods

We searched 15 international and national citation databases for population-level estimates on incidence rates of sepsis or severe sepsis per 100,000 person-years and case fatality rates in adult populations using consensus criteria and published in the last 40 years. No language or publication restrictions were applied. Studies were stratified into four subgroups (setting: hospital or ICU for sepsis and severe sepsis) and meta-analyzed using metaprop of the R 3.0.2 package. Heterogeneity of the underlying effects across studies was expressed by the estimated τ, the square root of the between-study variance.

## Results

The search yielded 1,553 reports from 1979 to 2013, of which 37 met our criteria and 33 provided data for meta-analysis. The included studies were from 15 high-income countries in North America, Europe, Asia, and Australia. For these countries, the population incidence rate was 256 (95% CI, 182 to 360, τ = 0.43) hospital-treated sepsis cases and 151 (95% CI, 94 to 242, τ = 0.98) hospital-treated severe sepsis cases per 100,000 person-years, with large between-study heterogeneity. Restricted to the last decade, the incidence rate was 427 (95% CI, 281 to 648, τ = 0.24) sepsis cases and 331 (95% CI, 207 to 530, τ = 0.59) severe sepsis cases per 100,000 person-years. Hospital mortality was 15% for sepsis and 25% for severe sepsis during this period of time. There were no population-level sepsis incidence estimates from lower income countries. A tentative extrapolation from high-income-country data suggests global estimates of 30.7 million sepsis and 23.8 million severe sepsis cases, with potentially six million deaths each year.

## Conclusion

Our analyses underline the urgent need to implement global strategies to monitor sepsis morbidity and mortality - especially in low-income and middle-income countries. For further epidemiological studies, more consistent and standardized methodological approaches are needed to reduce between-study heterogeneity. In particular, further research on sepsis coding using administrative data seems necessary to derive sensitive and specific sepsis case identifications.

